# Open Lip Schizencephaly Misdiagnosed as Paralytic Poliomyelitis in an 85‐Year‐Old: A Case Report

**DOI:** 10.1002/ccr3.70556

**Published:** 2025-06-18

**Authors:** Zihan Guo, Patricia D. Scripko, Alida M. Gertz

**Affiliations:** ^1^ Wellstar Douglas Medical Center ‐ Family Medicine Residency Program US; ^2^ Division of Neurology University of Maryland Medical System ‐ Capital Region US

**Keywords:** case report, misdiagnosis, open lip schizencephaly, paralytic polio

## Abstract

The variable clinical presentations of open lip schizencephaly can lead to misdiagnosis, as occurred in this case originally misdiagnosed as polio in the 1930s. This case demonstrates the possible presence of confirmation bias, as the original poliomyelitis diagnosis remained unquestioned despite developments in the fields of Radiology and Neurology.

**Abbreviations**
CT: Computerized tomography
MRI: Magnetic resonance imaging

## 1 Background

The differential diagnosis of hemiparesis at birth is broad and includes but is not limited to infection, congenital malformations, genetic and metabolic disorders, perinatal stroke, intracranial hemorrhage, peripartum injury, tumors, and neuromuscular disorders. Cerebral malformations are varied in anatomic location and severity of functional manifestation. Infections are also heterogeneous but could potentially include bacterial, viral, or even parasitic diseases. We present an 85‐year‐old woman with a history of dementia and left upper and lower extremity hemiparesis reportedly due to paralytic poliomyelitis.

## 2 Case Presentation

An 85‐year‐old female with a history of hemiparesis since birth presented to the emergency department with her daughter, who noted she had generalized weakness and fever for 4 days. According to the patient's daughter, her symptoms were associated with increased somnolence and difficulty getting out of bed. At baseline, she was reportedly able to ambulate without assistance despite her left‐sided hemiparesis and was alert and oriented to herself.

### 2.1 History

The patient's past medical history was limited to the collateral history provided by her daughter due to severe memory impairment secondary to Alzheimer's dementia. Before dementia onset, she had not exhibited signs of cognitive delay or other neurologic signs or symptoms aside from the left hemiparesis present since birth. She had told her daughter that the hemiparesis had not been progressive and was secondary to poliomyelitis infection. The patient had never been evaluated by a neurologist in adulthood or undergone brain imaging. The patient's daughter was unaware of any previous treatments or rehabilitation efforts for her mother's condition, nor did she report any additional associated symptoms. The patient was also not taking any chronic medications.

### 2.2 Diagnostic Process

**FIGURE 1:**
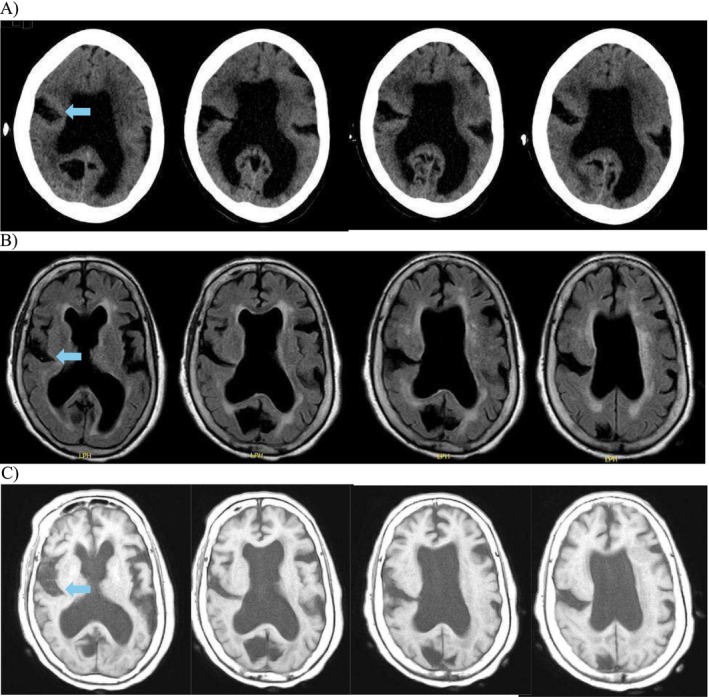
(A) Computerized tomography brain without contrast showing inferior to superior sequence (shown left to right) of right‐sided schizencephaly extending from the cortex to the right lateral ventricle. (B) Magnetic resonance imaging brain without contrast (axial T2 FLAIR) showing inferior to superior sequence (shown left to right) of right‐sided schizencephaly extending from the cortex to lateral ventricle and hypoplasia of the corpus callosum. (C) Magnetic resonance imaging brain without contrast (axial T1) showing margins of the schizencephaly defect lined with dysplastic gray matter. *Arrows indicate cleft extending from the right cerebral cortex into the lateral ventricle.

In the emergency department, the patient had vital signs within normal limits. She tested positive for SARS‐CoV‐2, first by antigen test and subsequently by PCR. A chest X‐ray showed no acute findings. SARS‐CoV‐2 infection was determined to be the cause of her presenting symptoms. A computerized tomography (CT) scan of the brain showed no signs of acute intracranial hemorrhage or infarction and was notable for advanced temporal lobe dominant cerebral atrophy, which correlated with her dementia. Incidentally, the CT revealed a possible right open lip schizencephaly with hypoplastic corpus callosum as depicted in Figure 1A–C which show evaluation by CT and magnetic resonance imaging (MRI) of the brain, confirming the presence of a cleft extending from the right cerebral cortex into the lateral ventricle, consistent with open lip schizencephaly.

The patient was admitted to the hospital by the Family Medicine inpatient team for overnight observation only given her advanced age. The patient was started on nirmatrelvir/ritonavir for SARS‐CoV‐2. The following morning, her fatigue and mobility returned to baseline. On examination by the Family Medicine team, the patient had left sided hemiparesis evidenced by a left upper motor neuron facial droop as well as 3/5 strength in her left upper and lower extremities. She had increased tone on the left and held her left upper extremity in a flexed posture, but was able to fully extend the affected limb. Her exam was limited by her dementia. Given her age, the progression of her cognitive dysfunction, and the stable nature of her hemiparesis, further evaluation and management in hospital were not pursued, and she was discharged home into the care of her daughter.

The case was later presented and reviewed by Family Medicine and Neurology. The weakness pattern was determined to be consistent with a right cortical lesion, fitting her right‐sided, open lip schizencephaly rather than paralytic poliomyelitis or post polio syndrome.

## 3 Discussion and Conclusions

### 3.1 Schizencephaly

Schizencephaly is a congenital brain malformation in which disordered neuronal migration causes one or more fluid‐filled clefts to form, connecting the cerebral hemisphere with the lateral ventricle [1]. The incidence of this condition is rare and is estimated to be between 0.54 and 1.54 per 100,000 live births in the United States and Europe [2, 3, 4, 5]. Diagnosis is generally made via imaging, and MRI is considered the modality of choice [1]. Clinically, two types of schizencephaly have been described. In type I, or closed schizencephaly, the edges of the clefts are fused. In type II, or open schizencephaly, the edges of the clefts are separated [6]. The etiology of schizencephaly is unknown and has been associated with many genetic and environmental factors. Symptoms and signs of schizencephaly can present at any age, including hemiparesis, spasticity, global developmental delay, seizures, microcephaly, and aplasia/hypoplasia of the corpus callosum [1]. In open lip schizencephaly, the malformation typically involves one cerebral hemisphere involving the frontal cortex and clinically manifests with paresis and plegia that do notprogressively worsen and fit a cortical pattern of weakness.

Although there has been some controversy over the definition of schizencephaly, within the past decade, an updated, specific definition has been advanced [7]. A recent but generally accepted definition states that it is transmantle column of dysplastic gray matter extending over the cerebrum or cerebellum from ependyma to the pia [8].

### 3.2 Poliomyelitis

Poliomyelitis and post‐polio syndrome (PPS) present with lower motor neuron syndrome and have a different pattern and course than schizencephaly. During the acute phase of paralytic poliomyelitis, poliovirus crosses the blood–brain barrier and causes anterior horn degeneration and apoptosis. These features present as asymmetrical descending flaccid paralysis associated with reduced tone and absent or reduced deep tendon reflexes on the affected side.

Adults who had paralytic poliomyelitis in childhood may later develop PPS [9]. PPS presents with progressive muscle weakness, muscle atrophy, and fasciculations that typically occur at least 15 years after the initial infection [9, 10]. These paralytic features of PPS are thought to be caused by the reinnervation of affected muscles following the acute phase of infection [10]. Despite the prevalence of poliovirus, PPS had no diagnostic criteria until 1991, which evolved to the current March of Dimes diagnostic criteria. These criteria are: (i) history of polio with neurologic symptoms; (ii) a period of recovery following initial illness and stabilized function; (iii) progressive weakness; (iv) fatigue and new symptoms that last for at least 1 year; and (v) exclusion of other more likely diagnoses [11, 12].

### 3.3 Our Patient

Our patient was born in the 1930s, before poliomyelitis and post polio syndrome were well understood. She was reportedly born with left‐sided hemiparesis and was diagnosed with paralytic poliomyelitis at birth. We now know that poliovirus cannot cross the fetal blood–brain barrier in utero, and neonates who contract the virus from affected mothers are exposed via olfactory and nerve endings after membrane rupture [12]. Our patient's mother's poliovirus status was unknown. However, had her mother been affected by poliovirus, the incubation period for paralytic disease is 7–21 days [13, 14] and is unlikely to cause hemiparesis at birth. Our patient was likely misdiagnosed with paralytic poliomyelitis due to medical limitations at the time, and it appears that the neurological deficits present at birth were due to cerebral malformations from open lip schizencephaly. Although the patient was unable to provide her perspective, her daughter was surprised to learn her mother's real diagnosis.

Open lip schizencephaly is a rare neuronal migration disorder. The wide range of variable and nonspecific potential clinical presentations of this condition can lead to misdiagnosis. These misdiagnoses could have been more prevalent before the advent of widely available advanced medical imaging. Paralytic poliomyelitis was accepted at the time of diagnosis due to the prevalence of the disease in the early twentieth century. A paralytic poliomyelitis diagnosis was not confirmed as medical advancements and diagnostic criteria were developed. With these developments, the original poliomyelitis diagnosis was unquestioned, possibly due to confirmation bias over decades. Physician assumptions due to the patient's age, along with a likely lack of awareness regarding schizencephaly, could have potentially contributed to the confirmation bias.

This case demonstrates that incidental findings from modern medical imaging can prompt questioning and correction of prior diagnoses.

## Author Contributions

**Zihan Guo:** conceptualization, data curation, investigation, resources, validation, writing – original draft, writing – review and editing. **Patricia D. Scripko:** investigation, supervision, writing – review and editing. **Alida M. Gertz:** conceptualization, methodology, project administration, resources, supervision, writing – review and editing.

## Ethics Statement

This case report was reported to the Wellstar research committee. No IRB determination was required.

## Consent

Written informed consent was obtained from the patient for publication of this case report and any accompanying images. A copy of the written consent is available for review by the Editor‐in‐Chief of this journal.

## Conflicts of Interest

The authors declare no conflicts of interest.

## Data Availability

All data gathered are reported in the paper.
